# Virulence Characteristics and Genetic Affinities of Multiple Drug Resistant Uropathogenic *Escherichia coli* from a Semi Urban Locality in India

**DOI:** 10.1371/journal.pone.0018063

**Published:** 2011-03-25

**Authors:** Savita Jadhav, Arif Hussain, Savita Devi, Ashutosh Kumar, Sana Parveen, Nageshwari Gandham, Lothar H. Wieler, Christa Ewers, Niyaz Ahmed

**Affiliations:** 1 Department of Microbiology, Dr. D. Y. Patil Medical College and Hospital, Pimpri, Pune, India; 2 Pathogen Biology Laboratory, Department of Biotechnology, School of Life Sciences, University of Hyderabad, Hyderabad, India; 3 Veterinary Faculty, Institute for Microbiology and Epizootics, Freie Universitaet Berlin, Berlin, Germany; 4 Institute of Biological Sciences, University of Malaya, Kuala Lumpur, Malaysia; 5 Institute of Life Sciences, University of Hyderabad Campus, Hyderabad, India; Cairo University, Egypt

## Abstract

Extraintestinal pathogenic *Escherichia coli* (ExPEC) are of significant health concern. The emergence of drug resistant *E. coli* with high virulence potential is alarming. Lack of sufficient data on transmission dynamics, virulence spectrum and antimicrobial resistance of certain pathogens such as the uropathogenic *E. coli* (UPEC) from countries with high infection burden, such as India, hinders the infection control and management efforts. In this study, we extensively genotyped and phenotyped a collection of 150 UPEC obtained from patients belonging to a semi-urban, industrialized setting near Pune, India. The isolates representing different clinical categories were analyzed in comparison with 50 commensal *E. coli* isolates from India as well as 50 ExPEC strains from Germany. Virulent strains were identified based on hemolysis, haemagglutination, cell surface hydrophobicity, serum bactericidal activity as well as with the help of O serotyping. We generated antimicrobial resistance profiles for all the clinical isolates and carried out phylogenetic analysis based on repetitive extragenic palindromic (rep)-PCR. *E. coli* from urinary tract infection cases expressed higher percentages of type I (45%) and P fimbriae (40%) when compared to fecal isolates (25% and 8% respectively). Hemolytic group comprised of 60% of UPEC and only 2% of *E. coli* from feces. Additionally, we found that serum resistance and cell surface hydrophobicity were not significantly (p = 0.16/p = 0.51) associated with UPEC from clinical cases. Moreover, clinical isolates exhibited highest resistance against amoxicillin (67.3%) and least against nitrofurantoin (57.3%). We also observed that 31.3% of UPEC were extended-spectrum beta-lactamase (ESBL) producers belonging to serotype O25, of which four were also positive for O25b subgroup that is linked to B2-O25b-ST131-CTX-M-15 virulent/multiresistant type. Furthermore, isolates from India and Germany (as well as global sources) were found to be genetically distinct with no evidence to espouse expansion of *E. coli* from India to the west or *vice-versa*.

## Introduction

Urinary tract infections (UTI) are the second most common human infections and are mainly caused by uropathogenic *E. coli.* The severity of UTI depends both on the virulence of the bacteria and the susceptibility of the host [1]. UPEC harbor numerous virulence factors including alpha-hemolysin, cytotoxic necrotizing factor, adhesins and iron acquisition systems. These factors support their ability to adhere to uroepethelial cells, help resist the bactericidal effect of serum and augment cell surface hydrophobicity thereby leading ultimately to tissue damage [2], [3], [4], [5].

Adherence to the urinary tract mucosa might protect bacteria from urinary lavage and in turn augment their ability to survive and invade renal tissues [6]. Specific adhesion is mediated by certain adhesins which can be differentiated based on their receptor binding specificity.

P fimbriae that are encoded by the *E. coli pap* (pyelonephritis-associated pilus) operon are the most important mannose-resistant adhesins, although they are expressed by only a limited number of *E. coli* serotypes. The most rampant P-fimbriated serotypes of the UPEC strains revealed one of the six O groups O1, O2, O4, O6, O7, and O18 [6], [7], [8].

Hemolysin production is another important virulence property of UPEC. Hemolysins inflict direct cytotoxic effects on renal epithelium resulting in scaring. Alpha-hemolysin is described to be a lethal factor with dermonecrotic effects and is antigenic in nature. Also, hemolysins are toxic to a series of host tissues and cells including RBCs, leucocytes, epithelial and endothelial cells. The frequency of isolation of hemolytic *E. coli* significantly associates with the severity of the infection [7], [8].

The importance of cell surface hydrophobicity as a virulence attribute (that facilitates bacterial adherence to mammalian cells) is known for nearly a century now, thanks to the pioneering studies of Mudd and Mudd [4]. It is an important factor helping *E. coli* to adhere to various surfaces for colonization. Bacteria are lysed by normal serum due to the activity of the complement system. The alternate pathway of complement activation is potentially important than the classical pathway. Bacterial resistance to killing by serum results from individual or combined effects of capsular polysaccharides, lipopolysaccharides and surface proteins [4]. Given this generic virulence ‘arsenal’ of UPEC, strains from different geographical regions pose different disease severity and should be genetically different.

To screen for the above mentioned virulence attributes and to find the most predominant serotypes among UPEC in the western Indian region, we analyzed 150 human clinical isolates. We intended to classify them epidemiologically into different serotypes and to obtain virulence marker profiles of UPEC (which have different frequencies in different disease conditions ranging from asymptomatic bacteriuria to chronic pyelonephritis). In addition, increasing antimicrobial resistance in bacterial pathogens is of major concern as it can vary according to geographical and regional situations [9], [10], [11], [12]. It is very relevant to ensure the appropriate therapy based on full knowledge of the organisms that cause UTI and their antibiotic susceptibility profiles. Therefore, it is necessary to do bacteriological testing also with reference to extended-spectrum beta-lactamase (ESBL) producers with resistance to beta-lactam antibiotics, including third generation cephalosporins such as cefotaxime, ceftriaxone and ceftazidime. Not much information on ESBL producing organisms causing UTI is available from India.

The present work was essentially carried out as an important precursor of a larger study aimed at understanding the transmission dynamics, population genetic structure and virulence mechanisms of UPEC from India. Herein, we present a much needed snapshot comprising of the virulence characteristics and antimicrobial resistance patterns of the available UPEC strains from western India representing the clinical conditions such as symptomatic UTI, bacteriuria, pyelonephritis, cystitis, prostatitis, septicemia and pyrexia of unknown origin (PUO). We sought to define the clinical correlates of different virulence factors and their association with various biological and life-style related factors of the host. In addition, we used rep-PCR based DNA profiling to know the genetic affinities of our isolates and to explore if this simple PCR based genetic analysis helps in understanding their spread patterns indicative of their diverse or clonal relationships. Also, we attempted to use this technique to know whether the bacterial isolates have any geographic inclination and how similar or different they are when compared to some of the well characterized isolates from western countries such as Germany.

## Materials and Methods

### Ethics statement

Written informed consents were obtained from all patients and healthy controls for the use of their strains which were cultured as part of compulsory diagnostic screening. Study protocols were approved by the institutional ethics committee of Dr. D. Y. Patil Medical College, Pune and by the Institutional Biosafety Committee of the School of Life Sciences of the University of Hyderabad, India.

### Study population and strains

One hundred and fifty *E. coli* isolates were obtained from urine samples of human patients in cell counts of 10^5^/ml. Fifty fecal *E. coli* isolates (that were used as controls), were isolated from the feces of healthy individuals who had reported for routine health checkup between January 2009–March 2010 at Dr. D. Y. Patil Medical College and Hospital, Pimpri, Pune, India. Patient background and provisional diagnosis of the infection were obtained from hospital records (Table S1). Identification of isolates was done using standard microbiological techniques [13], [14], [15]. All strains were stored on 15% glycerol-supplemented Luria-Bertani medium at −80°C.

### DNA samples

DNA samples of fifty ExPEC isolates from Germany and from global sources [16] were obtained; these represented various animal and human specimens, including 17 UPEC and archetypical newborn meningitis strain RS218 from humans, two UPEC from dogs and 31 avian pathogenic *E. coli* (APEC) from different birds. These strains were used for repetitive extragenic palindromic (rep) PCR together with the aforementioned human clinical isolates.

### Antimicrobial susceptibility test

The antimicrobial susceptibility testing was carried out on Mueller Hinton agar by disc diffusion method using the following antimicrobial substances [17]: amoxicillin (10 µg), ceftazidime (30 µg), ciprofloxacin (10 µg), co-trimoxazole (25 µg), gentamicin (10 µg), nitrofurantoin (300 µg), nalidixic acid (30 µg), and tetracycline (30 µg). Multi-drug resistant (MDR) strains were defined as those which showed resistance to three or more antimicrobial substances. Extended-spectrum beta-lactamase (ESBL) production was detected by the double-disk synergy (DDS) test as recommended by the Clinical and Laboratory Standards Institute (CLSI, 2006). Its presence was assayed using the antibiotic disks comprising of ceftazidime and ceftazidime/clavulanic acid (30/10 µg). An isolate was graded ESBL producer when its zone of inhibition varied by ≥5 mm among at least one of the combination disks and its coordinate comprising of standard antibiotic disk. *E. coli* ATCC 25922 was used as a negative control while *K. pneumoniae* ssp. *pneumoniae* ATCC 700603 served as positive control [18], [19].

### Phenotypic assays to determine virulence factors

#### a) Alpha-hemolysin production

The detection of alpha-hemolysin was performed by analysing the hemolytic zone observed after overnight growth at 37°C on sheep blood (5%) agar [20].

#### b) Haemagglutination

A suspension of human A +ve blood and PBS was mixed on a VDRL (venereal diseases research laboratory - test) cavity slide with a single colony of *E. coli*. After incubation on rotor at room temperature for some minutes, agglutination was seen. Similarly, haemagglutination was carried out in the presence of D-mannose. An ATCC *E. coli* 25922 strain was used as a negative control for mannose sensitive haemagglutination assay and a known strain of *E. coli* repeatedly giving positive was taken as control for the assay [21].

#### c) Cell Surface Hydrophobicity

Bacteria were tested for their hydrophobic property by using different molar concentrations of ammonium sulphate in VDRL tile; 40 µl of bacterial suspension in PBS was added in each of the wells containing 1 M, 1.4 M and 2 M ammonium sulphate. Clumps were seen by naked eyes. Strains were considered hydrophobic, if they aggregated in the PBS concentration of ≤1.4 M [21].

#### d) Serum Bactericidal Assay

Bacteria were diluted in Hank's balanced salt solution to get appropriate dilutions and mixed with human serum in a sterile tube and incubated in a water bath at 37°C and inoculated on nutrient agar plates at 0 hr, 1 hr, 2 hr of incubation in water bath. Growth at 0 hr was taken as control. *E. coli* was considered sensitive if count dropped to 1% and was considered resistant if >90% of the organisms survived after 2 hrs of incubation. An *E. coli* isolate that was consistently serum resistant and a one which was consistently sensitive were used as positive and negative controls respectively [22].

### Serotyping

Typing of somatic antigens was performed at the National Salmonella and *Escherichia* Centre, Central Research Institute, Kasauli, India, using antisera against O antigens - O1 to O173. O25 positive *E. coli* strains were further subjected to genotyping by a recent method based on allele-specific PCR targeting the *rfb*O25b subgroup gene locus [23].

### Repetive extragenic palindromic PCR

DNA sequences of primers used for Rep - fingerprinting were as follows: Forward Rep2a2: 5′-ACGGCTTATCGGGCCTA-3′, Reverse Rep1Ra1:5′-GCGACGGCATCAGGC-3′. PCR amplification was carried out in 20 µl reaction mixture consisting of 10× Taq buffer, 25 mM MgCl_2_, 10 mM dNTP, 100% DMSO, 100 pM of each primer and 2 U of Taq DNA polymerase and 5 µl of template. The PCR conditions were 95°C for 7 min, followed by 30 cycles of DNA amplification consisting of 45 s at 95°C, 1 min at 40°C and 8 min at 65°C followed by 16 min incubation at 65°C. The amplicons were run on 1.5% agarose gels and were analyzed by Bionumerics® software [24], [25].

### Detection of O25b subgroup strain by PCR

The newly described O25b O type *E. coli* were detected by using the following primers gndbis.f (5′ATACCGACGACGCCGATCTG-3′) and rfbO25b.r (5′TGCTATTCATTATGCGCAGC-3′). Annealing temperature of 60°C was used to generate a PCR product of 300 bp with the conditions as previously described [23].

### Statistical Analysis

Chi square test was used to compare the occurrence of virulence markers in cases and controls. P values less than 0.05 was considered significant.

## Results and Discussion

### Incidence of UTI in relationship with gender and socioeconomic status

In this study we observed a higher proportion of UTI in females (64%) than in males (36%). This is understandable due to the anatomy and is a consistent trend worldwide. Peak in the incidence of UTI was observed in the age groups 11–21 and 60–71 years. Among these, elderly patients are likely predisposed to conditions such as urinary tract obstruction, poor bladder emptying, and diabetes mellitus, etc. These factors favor colonization of bacteria and play an important role in UTI. Other studies have also reported similar findings [26], [27]. India has a large infection burden and the genito-urinary infections are very prominent. This may be due to a less affordable personal/community hygiene for some of the economically backword populations. We targeted these groups and analyzed strains obtained from such communities who reported for the UTI in periurban Pune. As such there is not much information available from India on the genetic and phenotypic diversity of UPEC. This information should therefore be construed as the first systematic analysis performed on a diverse type of patients/samples.

### Virulence characteristics of UPEC from India

#### a) Type 1 (MSHA) and P fimbriae (MRHA)

In the present study, 45% of the UTI isolates and 25% of fecal isolates showed the presence of type 1 (Mannose sensitive hemagglutination - MSHA) fimbriae. Thus the difference among UTI and fecal isolates was not statistically significant. It was found that 40% of UTI isolates and 8% of *E. coli* isolates from the controls exhibited P fimbriae (Mannose resistant hemagglutination -MRHA). The difference was statistically significant (P = 0.0002). Expression of type 1 fimbriae was more discerned in isolates from the cases of simple UTI and cystitis. In our observation, maximum P fimbriae positive isolates were highly associated with simple UTI, while only 10% of such isolates caused pyelonephritis. As such there is no relation among the occurrence or severity of symptoms/the site of infection and fimbrial expression (of P or type1) in case of bacterial isolates from urine [28].

Our observation was in agreement with another study [29], wherein the presence of type 1 fimbriae was seen in 71% of isolates of *E. coli* from UTI and in 60% of isolates of *E. coli* from the control fecal flora. Taken together, UTI associated isolates did not show any significant expression of MSHA compared to controls; however, Najar *et al.* (2007) [29] showed an overall higher expression of type 1 fimbriae in both the UTI isolates and controls. Many studies have demonstrated the presence of P fimbriae as maximum in UTI isolates than in fecal isolates of healthy persons [29], [30].

Although, since we looked only at phenotypes of the isolates *in vitro*, it is difficult to dwell upon the real significance of the fimbrial expression patterns *vis a vis* the niche tropism (upper or lower UTI) and its impact on different outcomes of infection – these are largely the phenomena due to microbial mechanisms operating *in vivo*. Normally, the expression of P fimbriae is increased among isolates associated with lower urinary tract infections, basically resembling an ‘*in vivo*’ situation as there is an interplay between type1-fimbriae and P-fimbriae expression (“switch-off” of Type 1 fimbriae leads to increased motility of bacteria and finally to binding of P-fimbrial adhesin to receptors on kidney epithelial cells). The higher or lower expression itself might therefore only give an idea about the quantitative occurrence of P fimbriae, but not about the different adhesion subunits which would be more relevant with respect to upper/lower UTI infections.

#### b) Hemolysin

Production of alpha hemolysin was observed in 60% of UPEC from the cases while only 2% of *E. coli* isolates from feces produced alpha hemolysin (P = 0.0001). Other studies have also reported similar findings [31]. In our observation, hemolysin was highest in pyelonephritis, lower in cystitis and least in asymptomatic bacteriuria.

#### c) Serum resistance and cell surface hydrophobicity

Serum resistance and cell surface hydrophobicity markers were not significantly present in *E. coli* isolates from patients compared to control fecal isolates: 55% and 76% of urinary *E. coli* isolates showed serum resistance and cell surface hydrophobicity respectively and the control isolates showed 40% and 70% for serum resistance and cell surface hydrophobicity respectively.

The difference in both the cases was not statistically significant (p = 0.16/p = 0.51). This trend has been reported earlier and our study confirmed the same for Indian isolates [32].

Occurrence of virulence factors in UPEC strains confirms the association of UPEC with urinary pathogenicity. It was interesting to note that UPEC with virulence factors were significantly more prevalent in patient groups than in controls (Table 1). Such a significant difference was noticed earlier, indicating that several virulence factors act synergistically to cause infection of the urinary tract [33]. Nevertheless, it was interesting that 8% of the isolates (12/150) from infection cases did not reveal any virulence markers under investigation. These were the isolates from complicated UTI. It appears that these isolates, though devoid of any virulence factors as probed by us, were able to localize in the deeper tissues of a compromised urinary tract where scarcely any drug reaches in therapeutic quantum. It may also be due to the reason that they might possess virulence markers/mechanisms other than those tested by us.

**Table 1:** Occurrence of virulence phenotypes in *E. coli* isolates from cases and controls.

Virulence phenotypes	Number of isolates from cases (%) total cases = 150	Number of isolates from controls (%) total controls = 50	P-value
MRHA	60 (40.0)	4 (8.0)	0.0002
MSHA	68 (45.3)	12 (24.0)	0.406
Hemolysin	90 (60.0)	1 (2.0)	0.00001
SR	83 (55.3)	20 (40.0)	0.16
CSH	120 (80.0)	35 (70.0)	0.51

We screened 19 mucoid UPEC isolates by phenotypic and biochemical characterization and found that all were from patient groups and were capsulated. Out of the 19 mucoid UPEC, 11 were capsulated and serum resistant. Capsule confers serum and phagocyte resistance and this could be attributed to sialic acid residues that subvert the ability of bacterial surface to activate complement by alternative pathway thereby augmenting the virulence potential of such pathogens. However, eight of the mucoid strains were susceptible to serum, perhaps due to the non capsular factors and this might have a role in serum resistance.

### Antimicrobial susceptibility profiles of UPEC

**Figure 1:**
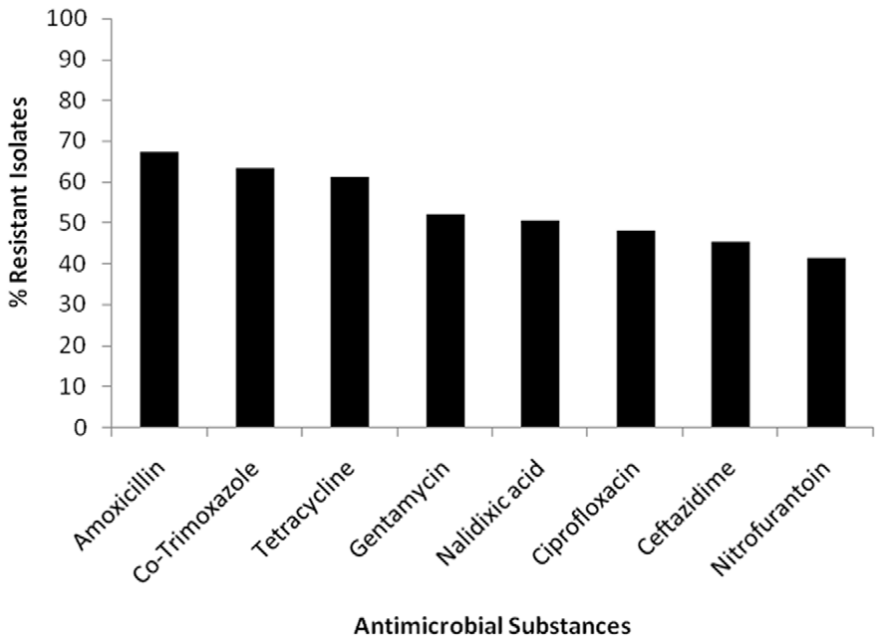
The antimicrobial susceptibility and resistance pattern of 150 UTI isolates from India. Nitrofurantoin was consistently the most active (59%) of the systemically active antimicrobials, with ceftazidime, giving similar results (54.7%). These were followed by ciprofloxacin (52%), nalidixic acid (49%) and gentamicin (48%), but sensitivity to amoxicillin was found to be low (32.7%). Sensivity profiles of Co-trimoxazole (36.7%) and tetracycline (38.7%) were quite similar.

**Figure 2:**
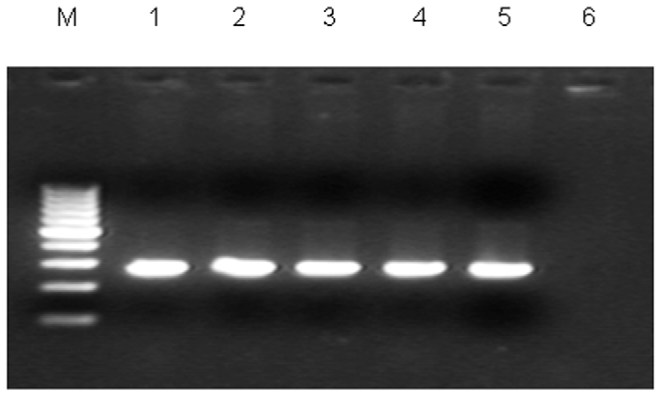
PCR based detection of O25b subgroup that is linked to the B2-O25b-ST131-CTX-M-15 clonal group of strains. Among 32 ESBL producers in our collection (n = 150) four were positive for O25b subgroup (Lanes 1 to 4); lane 5, positive (strain showing consistently positive); lane 6, negative; M, 100 bp DNA ladder.

Antimicrobial susceptibility testing was carried out on all the clinical isolates. The majority of isolates were sensitive to nitrofurantoin - 86 (57.3%) followed by ciprofloxacin - 78 (52%) and nalidixic acid - 74 (49%). This study showed high resistance to amoxicillin - 101 (67.3%), tetracycline - 92 (61.3%), and cefotaxime - 68 (45.3%) (Figure 1). Upon testing for ESBL, we found that 32 (21.3%) were ESBL producers. A high proportion (31.3%) of these ESBL-producing isolates was belonging to the serotype O25. Four of these were positively tested for the subgroup O25b (Figure 2), which has been linked to a clonally related group of highly virulent, multiresistant *E. coli* strains (B2-O25b-ST131-CTX-M-15) that are emerging among humans and animals worldwide [34], [35]. Further studies, i.e. multi locus sequence typing (MLST), macrorestriction analysis, and determination of beta-lactamase enzymes are needed to unravel the phylogenetic relatedness of these O25-ESBL-producing Indian isolates to the worldwide recognized clonal groups and to characterize the remaining multiresistant isolates observed. ESBL isolates were frequently (65.6%) associated with a hemolytic phenotype, with a higher rate than the non-ESBL-producing UPEC strains (58.5%). Hence, although it is a commonly accepted fact that bacterial pathogens acquire resistance determinants and express a multiresistant phenotype at the cost of their virulence properties, the frequent observation of hemolytic UPEC among our strain collection underlines the possible emergence of highly virulent multiresistant strains [36] in this area.

It is particularly worrisome that more than half of the ESBL producers (53.1%) in our collection were resistant to ciprofloxacin suggesting that they may be resistant to all available fluoroquinolones - the drug of choice for treatment of infections caused by ESBL-producing strains. Overall, nitrofurantoin and ciprofloxacin were found superior to amoxicillin and co-trimoxazole. None of the uropathogens from this study area were 100% susceptible to any of the antimicrobial substances used. Prior studies have shown a sensitivity rate of 95–100% to nitrofurantoin [37]. But in our study, nitrofurantoin showed only 57.3% sensitivity; the reason for this low sensitivity and high resistance to almost all antimicrobials in the study area may indicate difference in antimicrobial usage, infection control practices and other unrecognized factors including genetic propensity of these strains to accumulate mutations conferring MDR phenotypes. Periodic review and formulation of antibiotic policy are needed to control acquisition of drug resistance. Further studies for better understanding of the interaction of different virulence factors at molecular level are necessary as most urovirulent strains express multiple virulence factors simultaneously. We believe that the methods of detection of the above mentioned virulence markers are reasonably easy and screening them in a clinical microbiology laboratory is a worthwhile exercise.

### Serotyping and genotyping

In our study, as many as 19 different serotypes (O1, O2, O9, O14, O20, O25, O44, O45, O60, O64, O76, O79, O84, O102, O116, O120, O130, O95, O100) were observed among the cultured UPEC. Out of these, 27 *E. coli* were untypeable; six expressed a rough O antigen and five were *Escherichia* species (non- *E. coli*; NEC). A majority of *E. coli* isolates expressed O antigens - O25 (31.9%), O102 (9.7%), O120 (8.3%), and O1 (5.6%). Serotype O25 was predominantly found to be isolated from patients with pyelonephritis, prostatitis, cystitis and simple UTI. Renal failure associated isolates mainly belonged to O25 and O102 serotypes (Table 2).

**Table 2:** Distribution of various O-antigens in clinical isolates of *E. coli*.

O-Antigen	Pyelonephritis	Prostatitis	Cystitis	Simple UTI	Septicaemia	PUO	Total
O1	-	1	-	5	2	-	8
O2	-	1	-	1	-	-	2
O9	-	-	-	-	-	-	0
O14	1	-	-	-	-	-	1
O20	1	-	-	-	1	1	3
O25	6	7	11	15	2	4	45
O44	1	-	-	2	1	-	4
O45	-	-	1	-	-	-	1
O60	1		1	4	1	-	7
O64	-	-	1	1	-	-	2
O76	-	-	1	3	-	-	4
O79	-	1	-	-	-	-	1
O84	-	-	-	1	-	-	1
O102	3	1	-	8	-	1	13
O116	-	-	-	1	-	-	0
O120	2	2	3	6	-	1	14
O130	-	1	-	1	-	-	2
Rough	1		1	2	2	-	6
O 95	1	-	-	-	-	-	1
O100	-	-	-	1	-	-	1
Non- viable	-		1		-	-	1
Non *E. coli*	-	1	1	2	-	1	5
Untypeable	5	-	3	13	3	4	28
Total	22	15	24	65	12	12	150

**Figure 3:**
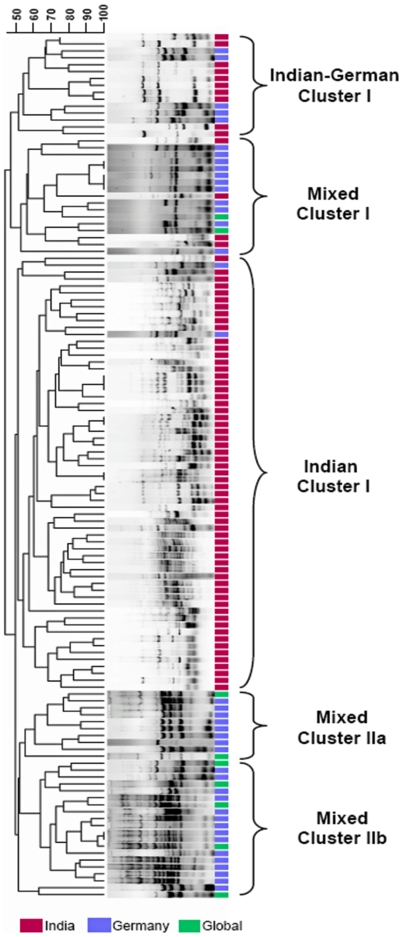
Dendrogram based on rep-PCR, developed in Bionumerics® revealed genetic relationships of *E. coli* representing Indian clinical isolates and ExPECs from global sources. Different geographic clusters are labeled. Upper scale denotes genetic distance.

To determine the relatedness of the strains, a dendrogram based on rep-PCR based fingerprint data was constructed in Bionumerics®. For this study, the clinical strains from India and 50 German isolates were used while the fecal isolates were excluded as the aim of this experiment was not to prove pathogenicity of the ExPEC but to see how diverse and clonal they are and to analyze Indian isolates in juxtaposition with German strains. Apart from the occurrence of singleton strains, the dendrogram obtained could be broadly classified into 5 major clusters: Indian-German cluster I (n = 15 strains), Indian cluster I (n = 63), mixed cluster I (n = 17) and mixed clusters IIa/IIb (n = 30), which include strains from all the three locales, as depicted in Figure 3. All the strains that clustered under Indian branch (exceptionally harboring one isolate from Germany) revealed very diverse pattern, except for a few ones - it appears that the bacteria we cultured were very diverse and the ones which were close in the cluster have no phenotypic resemblances with each other in terms of antimicrobial resistance profile or serotype or other virulence markers. This may be due to the reason that the UPEC in this study area could be highly diverse at sub-species and serotype levels.

The mixed cluster I contained a group of 17 bacterial isolates from India, Germany and from global sources. This cluster predominantly comprises of APEC from cases of septicemia in chicken or goose, while the remaining seven strains were human UPEC, including strain CFT073, or they were septicemia-associated strains (SEPEC). The mixed cluster II was subdivided into two subclusters- subcluster IIa (n = 10) and subcluster IIb (n = 20). The subcluster IIa largely encompasses APEC. Eight out of ten members of this cluster were APEC and the rest were UTI pathogens from humans. In the subcluster IIb, human isolates as well as isolates from animals were grouped together. There was no discrete band pattern between strains isolated from different sources; out of twenty strains in subcluster IIb nine were APEC, seven were UPEC of humans and two from dog, one was a human SEPEC and finally the archetypical NMEC strain RS218 grouping within this subcluster. From this analysis, we may conclude that the technique of rep-PCR is not resolving enough to distinguishing strains that portray similar phenotypic markers; nevertheless the Indian ExPEC population was clearly distinguishable from German ExPEC. We also found that the isolates from different animal sources were more or less grouping into distinct clusters, indicating that this technique can also be used to find the sources of infection in a reservoir such as a water body.

In conclusion, our observations form an important baseline data-set towards understanding the virulence properties, antibiotic resistance profiles and genetic diversity of UPEC from India. We hope that such observations will be more meaningful towards systematically unraveling the population genetic structure of UPEC and their propensity to spread, or emerge with multi drug resistance phenotypes in new epidemiological territories. In the backdrop of this work, future studies involving decipherment of MDR mechanisms, lineage tracking based on MLST [16] and whole genome sequencing of representative isolates from different MLST clades [2] shall be imminent. Further, the ‘omics’ inspired studies will ultimately help understanding of the functional molecular epidemiology and infection biology of Indian UPEC at greater details. On the clinical side, it will be possible to ascertain reservoirs of infection which could be important in understanding the mechanisms of chronic and recurrent UTI [38] in a community setting and how they are maintained within or in the vicinity of different human populations of a multicultural and densely populated country such as India.

## Supporting Information

Table S1 Clinical types, antibiogram, serotype and virulence characteristics of the UPEC isolates from Pune, India.
(DOC)
