# Supplementary material for: Virulence Characteristics and Genetic Affinities of Multiple Drug Resistant Uropathogenic Escherichia coli from a Semi Urban Locality in India
Source: PLoS One. 2011 Mar 25;6(3):e18063. doi: 10.1371/journal.pone.0018063 (PMC3064663; doi:10.1371/journal.pone.0018063)
Supplement: Table S1 — Clinical types, antibiogram, serotype and virulence characteristics of the UPEC isolates from Pune, India. (DOC) [file pone.0018063.s001.doc]

| **Table S1: Clinical types, antibiogram, serotype and virulence characteristics of the UPEC isolates from Pune, India**  isolate no. | patient age  (Yr) | sex | clinical diagnosis | antibiotic sensitivity  (1) | | | | | | | | serotype | MSHA | MRHA | | HLY | | SR | CSH | | ESBL | | |
| --- | --- | --- | --- | --- | --- | --- | --- | --- | --- | --- | --- | --- | --- | --- | --- | --- | --- | --- | --- | --- | --- | --- | --- |
|  |  |  |  | CF | NF | Ca | A | NA | T | CO | G |  | (2) | (3) | (4) | | (5) | | | (6) | | | (7) |
| 1 | 69 | F | Pyelonephritis | R | S | R | R | R | R | S | S | O14 | + | - | + | | - | | | + | | | + |
| 2 | 70 | F | Pyelonephritis | R | R | R | R | S | R | R | R | O25 | + | - | + | | - | | | + | | | + |
| 3 | 30 | F | Cystitis | R | S | R | R | S | S | R | S | NEC | - | + | - | | - | | | - | | | - |
| 4 | 20 | F | UTI | S | S | S | R | S | S | R | R | O76 | - | + | - | | + | | | + | | | - |
| 5 | 5 | M | UTI | R | S | S | S | R | R | S | S | O64 | + | - | - | | + | | | + | | | - |
| 6 | 40 | F | Cystitis | R | S | S | R | S | R | R | R | O76 | + | - | - | | + | | | + | | | - |
| 7 | 19 | F | UTI | S | S | S | S | R | S | S | S | O116 | + | - | - | | + | | | + | | | - |
| 8 | 52 | F | Cystitis | R | R | R | R | S | R | R | R | O2 | + | - | - | | + | | | - | | | + |
| 9 | 20 | M | UTI | S | S | S | S | S | S | S | S | O60 | + | - | - | | + | | | + | | | - |
| 10 | 2 | F | Septicemia | S | S | S | S | S | R | S | R | O44 | + | - | + | | - | | | + | | | - |
| 11 | 17 | M | Cystitis | S | S | S | S | R | S | S | S | O45 | + | - | + | | + | | | + | | | - |
| 12 | 30 | F | UTI | R | S | R | S | R | S | R | R | O44 | + | + | + | | + | | | + | | | - |
| 13 | 52 | M | Prostatitis | R | S | R | R | S | R | R | R | O120 | - | - | - | | - | | | + | | | + |
| 14 | 80 | M | Prostatitis | S | R | R | R | R | R | R | R | NEC | - | - | - | | + | | | - | | | - |
| 15 | 21 | M | UTI | S | S | S | R | R | S | S | R | O120 | + | + | + | | + | | | + | | | - |
| 16 | 18 | M | Cystitis | S | R | S | S | R | R | S | R | O120 | + | - | + | | + | | | - | | | - |
| 17 | 27 | F | UTI | S | S | S | R | S | R | S | S | O25 | - | + | + | | - | | | + | | | - |
| 18 | 10 | M | Septicemia | S | S | S | S | S | S | S | S | O25 | + | - | + | | - | | | + | | | - |
| 19 | 35 | F | UTI | R | R | R | R | R | R | S | S | UT | - | + | + | | + | | | + | | | + |
| 20 | 28 | M | UTI | R | R | R | R | R | R | R | S | O25 | - | - | + | | - | | | + | | | - |
| 21 | 21 | M | UTI | R | S | R | R | R | R | S | S | O102 | - | + | + | | + | | | + | | | + |
| 22 | 5 | M | UTI | S | S | R | I | S | S | S | R | O60 | - | + | + | | + | | | + | | | - |
| 23 | 60 | M | Prostatitis | R | R | R | R | S | R | R | S | O130 | + | - | + | | + | | | + | | | - |
| 24 | 38 | F | UTI | S | S | R | I | S | R | R | R | O120 | + | + | + | | + | | | + | | | - |
| 25 | 65 | M | Prostatitis | R | S | I | R | R | R | R | R | O130 | + | - | + | | + | | | + | | | - |
| 26 | 55 | M | Prostatitis | S | R | R | S | R | R | R | R | O25 | - | - | - | | - | | | + | | | + |
| 27 | 11 | F | UTI | S | S | S | R | R | S | R | S | O60 | - | + | + | | + | | | + | | | - |
| 28 | 56 | F | UTI | S | S | I | R | S | R | R | S | NEC | - | + | - | | - | | | - | | | - |
| 29 | 85 | M | Prostatitis | I | R | R | R | R | R | R | S | O9 | + | - | + | | + | | | + | | | - |
| 30 | 7 | F | UTI | S | S | I | R | S | R | S | R | O25 | + | + | - | | + | | | + | | | - |
| 31 | 20 | F | UTI | S | S | S | R | R | R | R | S | O25 | + | + | - | | + | | | + | | | - |
| 32 | 80 | F | Cystitis | R | R | R | R | S | R | R | R | O120 | + | - | + | | - | | | + | | | + |
| 33 | 56 | M | UTI | R | R | R | R | S | S | R | S | UT | + | + | - | | + | | | + | | | - |
| 34 | 65 | M | Prostatitis | R | R | R | R | S | R | S | R | O25 | + | - | - | | + | | | + | | | - |
| 35 | 65 | M | Septicemia | I | R | R | R | R | R | R | S | O1 | + | - | + | | - | | | + | | | - |
| 36 | 65 | F | Septicemia | R | R | R | R | R | R | R | R | O1 | + | - | - | | + | | | + | | | - |
| 37 | 67 | F | UTI | R | R | R | R | R | S | R | R | O120 | + | + | + | | + | | | + | | | - |
| 38 | 20 | F | UTI | S | S | S | S | S | R | S | R | O1 | - | + | + | | + | | | + | | | - |
| 39 | 35 | F | UTI | S | S | S | S | S | R | R | R | UT | - | + | - | | + | | | + | | | - |
| 40 | 1 | F | Septicemia | S | S | S | S | S | S | S | R | ROUGH | - | - | - | | - | | | + | | | - |
| 41 | 32 | M | Cystitis | R | S | I | R | S | S | S | S | O25 | - | - | - | | - | | | + | | | - |
| 42 | 60 | F | Pyelonephritis | R | R | R | R | R | R | R | S | O20 | + | - | + | | + | | | + | | | - |
| 43 | 42 | F | Pyelonephritis | R | R | I | R | R | R | R | S | O120 | + | + | + | | - | | | - | | | - |
| 44 | 50 | F | PUO | R | R | R | R | R | R | R | R | O120 | + | - | + | | + | | | + | | | + |
| 45 | 2 | M | PUO | S | S | S | S | S | S | S | R | UT | + | - | + | | - | | | - | | | - |
| 46 | 70 | F | Pyelonephritis | R | R | R | R | S | S | R | R | O102 | + | - | + | | + | | | + | | | - |
| 47 | 60 | F | Cystitis | R | R | R | R | S | S | R | S | UT | - | - | - | | - | | | + | | | - |
| 48 | 45 | F | UTI | S | S | S | R | S | R | R | S | ROUGH | + | + | - | | - | | | + | | | - |
| 49 | 2 | F | UTI | S | S | R | R | R | R | S | R | O120 | - | + | + | | + | | | + | | | + |
| 50 | 33 | F | UTI | S | S | S | R | R | R | R | R | O1 | + | + | + | | + | | | + | | | - |
| 51 | 64 | F | UTI | R | S | S | R | R | R | R | R | ROUGH | - | + | - | | - | | | + | | | - |
| 52 | 9 | F | UTI | R | R | S | R | S | R | S | R | O102 | + | - | + | | + | | | + | | | - |
| 53 | 60 | F | Pyelonephritis | R | R | R | R | S | S | R | R | ROUGH | - | - | - | | - | | | + | | | + |
| 54 | 80 | M | Prostatitis | S | R | S | S | S | R | R | S | O25 | + | - | + | | - | | | + | | | - |
| 55 | 72 | M | Prostatitis | S | S | S | R | S | S | R | S | O25 | + | - | - | | - | | | + | | | - |
| 56 | 40 | F | Septicemia | R | S | S | I | R | R | R | S | UT | + | - | - | | + | | | + | | | - |
| 57 | 65 | F | Pyelonephritis | R | R | S | R | R | S | S | R | UT | + | - | + | | - | | | + | | | - |
| 58 | 17 | M | Cystitis | R | S | S | S | R | R | S | S | UT | + | - | + | | - | | | + | | | - |
| 59 | 75 | F | Pyelonephritis | R | R | R | R | S | S | R | R | UT | - | - | + | | - | | | - | | | + |
| 60 | 23 | F | UTI | S | S | S | R | R | S | R | S | O25 | - | + | + | | + | | | + | | | - |
| 61 | 24 | F | UTI | S | S | S | S | S | R | R | R | O120 | - | + | - | | + | | | + | | | - |
| 62 | 20 | M | UTI | S | S | S | S | R | R | R | S | O25 | - | - | + | | - | | | - | | | - |
| 63 | 19 | F | UTI | S | S | R | S | S | R | R | R | O25 | - | + | - | | - | | | + | | | - |
| 64 | 61 | F | Pyelonephritis | R | R | R | R | S | S | R | S | O102 | + | - | + | | + | | | + | | | + |
| 65 | 10 | F | UTI | S | S | S | S | R | S | S | S | O25 | - | + | + | | + | | | + | | | - |
| 66 | 20 | F | UTI | S | S | S | R | R | S | S | R | UT | - | - | + | | - | | | - | | | - |
| 67 | 40 | F | UTI | S | S | R | S | S | R | R | R | UT | - | + | + | | + | | | + | | | + |
| 68 | 45 | F | Cystitis | S | R | R | R | R | R | R | R | UT | - | - | - | | + | | | - | | | - |
| 69 | 12 | F | UTI | S | S | S | R | S | S | S | R | O25 | - | + | - | | + | | | + | | | - |
| 70 | 12 | F | UTI | S | S | S | R | R | R | S | S | UT | - | + | + | | - | | | + | | | - |
| 71 | 72 | M | Prostatitis | R | R | R | S | S | S | R | S | O79 | + | - | + | | + | | | - | | | - |
| 72 | 70 | M | Pyelonephritis | R | R | R | S | S | R | S | S | UT | + | - | + | | - | | | + | | | + |
| 73 | 32 | M | UTI | S | S | R | R | R | R | R | S | O25 | + | - | + | | + | | | + | | | - |
| 74 | 60 | F | Cystitis | R | R | S | S | R | S | S | R | O25 | + | - | + | | + | | | - | | | - |
| 75 | 16 | F | Cystitis | S | S | R | R | R | S | R | R | O25 | - | - | - | | - | | | + | | | + |
| 76 | 74 | F | Pyelonephritis | S | R | R | S | S | S | S | S | UT | + | - | + | | - | | | - | | | - |
| 77 | 17 | M | PUO | R | S | S | S | S | R | R | R | UT | - | + | + | | + | | | + | | | - |
| 78 | 70 | F | Cystitis | R | R | S | S | S | R | S | S | O25 | - | - | - | | - | | | + | | | - |
| 79 | 12 | M | Cystitis | S | S | S | S | R | R | R | R | O25 | + | - | - | | + | | | + | | | - |
| 80 | 75 | F | Pyelonephritis | R | R | S | S | S | R | S | S | O25 | - | + | + | | - | | | + | | | - |
| 81 | 21 | F | UTI | S | S | R | S | R | R | R | R | O25 | - | + | + | | + | | | + | | | + |
| 82 | 40 | M | UTI | S | R | R | S | S | S | R | S | UT | - | + | - | | + | | | + | | | - |
| 83 | 65 | M | Prostatitis | S | S | R | R | R | R | S | R | O25 | + | + | + | | + | | | + | | | + |
| 84 | 19 | F | UTI | R | S | S | R | R | S | R | S | O25 | - | - | - | | + | | | + | | | - |
| 85 | 55 | F | UTI | R | S | S | R | R | R | R | S | O25 | - | + | + | | - | | | + | | | - |
| 86 | 61 | F | UTI | S | R | S | R | S | S | S | S | O25 | - | + | + | | - | | | - | | | - |
| 87 | 11 | F | UTI | S | R | R | R | S | R | S | S | UT | - | - | - | | + | | | + | | | - |
| 88 | 23 | M | UTI | S | S | S | R | R | S | R | S | O25 | - | + | - | | - | | | + | | | - |
| 89 | 70 | F | Cystitis | S | S | R | R | S | R | S | S | O60 | + | - | + | | - | | | + | | | + |
| 90 | 10 | M | Septicemia | I | S | S | R | R | S | R | R | O60 | + | - | + | | - | | | + | | | - |
| 91 | 65 | M | UTI | R | S | I | S | S | R | R | R | O102 | - | + | - | | + | | | - | | | - |
| 92 | 45 | F | UTI | S | S | R | S | R | R | R | S | O120 | - | + | + | | + | | | + | | | + |
| 93 | 50 | F | Pyelonephritis | R | R | S | S | S | R | S | R | O25 | - | - | - | | - | | | + | | | - |
| 94 | 16 | F | UTI | R | S | I | R | S | R | S | S | O25 | - | + | - | | + | | | + | | | - |
| 95 | 38 | M | Cystitis | R | R | S | R | S | R | S | R | O25 | + | - | - | | + | | | - | | | - |
| 96 | 22 | M | UTI | S | S | R | S | S | R | R | S | O44 | - | + | + | | + | | | + | | | - |
| 97 | 29 | M | Septicemia | S | S | R | S | S | R | R | R | ROUGH | - | - | - | | - | | | + | | | + |
| 98 | 40 | F | UTI | S | R | R | R | R | R | S | S | O1 | - | - | + | | + | | | + | | | - |
| 99 | 2 | F | Septicemia | R | S | S | R | R | S | S | R | UT | - | + | + | | + | | | - | | | - |
| 100 | 45 | F | Septicemia | R | S | S | R | S | S | R | S | UT | - | + | + | | + | | | - | | | - |
| 101 | 20 | M | Pyelonephritis | R | S | R | R | S | S | S | R | O25 | - | - | + | | + | | | + | | | + |
| 102 | 44 | M | UTI | S | R | I | R | S | R | R | R | UT | - | + | + | | - | | | + | | | - |
| 103 | 45 | F | UTI | S | R | S | R | R | S | S | R | O102 | - | - | + | | - | | | + | | | - |
| 104 | 5 | F | PUO | S | S | S | R | R | R | S | R | O20 | + | - | - | | + | | | + | | | - |
| 105 | 10 | F | PUO | R | S | S | S | R | S | S | S | O25 | + | - | - | | + | | | - | | | - |
| 106 | 55 | M | UTI | S | R | R | R | R | R | R | S | O60 | - | + | + | | + | | | + | | | - |
| 107 | 61 | F | UTI | S | R | R | S | S | S | S | S | UT | - | + | - | | + | | | + | | | + |
| 108 | 10 | F | PUO | I | S | S | S | S | R | R | R | NEC | - | - | - | | - | | | + | | | - |
| 109 | 65 | F | Cystitis | R | R | R | R | S | S | R | S | O25 | + | - | + | | - | | | + | | | - |
| 110 | 43 | F | PUO | R | R | S | R | R | R | R | R | O25 | - | + | - | | + | | | + | | | - |
| 111 | 60 | F | Pyelonephritis | R | R | S | R | R | R | S | S | O25 | - | - | + | | - | | | + | | | + |
| 112 | 12 | F | PUO | R | S | S | S | R | S | R | R | O25 | - | + | + | | + | | | - | | | - |
| 113 | 27 | M | PUO | S | S | S | R | R | R | R | R | O25 | - | - | + | | + | | | + | | | - |
| 114 | 70 | M | Prostatitis | S | R | R | R | S | S | R | R | O25 | - | - | - | | + | | | + | | | + |
| 115 | 72 | M | Prostatitis | S | R | R | R | S | S | R | R | O25 | + | - | - | | - | | | + | | | - |
| 116 | 60 | M | Pyelonephritis | R | R | S | R | R | S | R | S | O25 | - | + | + | | + | | | + | | | - |
| 117 | 56 | F | Cystitis | R | R | S | R | R | R | R | R | O25 | + | - | - | | - | | | - | | | - |
| 118 | 4 | M | PUO | S | S | S | R | R | R | R | S | UT | - | + | + | | + | | | - | | | - |
| 119 | 46 | F | Cystitis | R | R | S | R | R | R | R | S | O64 | + | - | + | | + | | | + | | | - |
| 120 | 65 | M | Pyelonephritis | R | R | S | R | S | S | S | S | O44 | - | + | + | | + | | | - | | | - |
| 121 | 26 | F | Cystitis | R | S | S | R | R | R | S | R | O25 | + | - | - | | + | | | + | | | - |
| 122 | 26 | F | UTI | S | S | S | R | S | S | S | S | O1 | - | + | + | | + | | | + | | | - |
| 123 | 56 | F | Septicemia | S | R | R | R | R | R | R | R | O25 | + | - | - | | + | | | + | | | + |
| 124 | 20 | F | UTI | S | S | R | R | S | R | R | S | NEC | - | - | - | | - | | | + | | | - |
| 125 | 35 | F | UTI | S | S | I | R | R | S | R | R | O76 | - | + | + | | + | | | + | | | - |
| 126 | 46 | F | Septicemia | R | R | R | S | S | R | R | S | O20 | - | + | + | | - | | | + | | | + |
| 127 | 29 | F | Cystitis | S | S | R | R | S | R | R | R | O25 | + | - | - | | + | | | + | | | - |
| 128 | 68 | F | Pyelonephritis | R | R | R | R | R | R | R | S | O60 | - | + | + | | - | | | + | | | + |
| 129 | 39 | F | UTI | S | S | S | R | R | S | R | R | O2 | + | + | + | | - | | | + | | | - |
| 130 | 34 | F | UTI | S | S | R | R | R | R | R | S | O84 | + | - | + | | - | | | + | | | - |
| 131 | 25 | M | Cystitis | R | S | S | R | R | S | S | S | O120 | + | - | + | | + | | | + | | | - |
| 132 | 1 | M | PUO | S | S | S | R | S | R | R | S | UT | - | - | + | | - | | | + | | | - |
| 133 | 21 | M | UTI | S | S | R | S | R | S | S | S | O76 | - | - | - | | + | | | - | | | + |
| 134 | 21 | F | UTI | S | R | R | S | R | R | R | R | UT | - | - | + | | + | | | + | | - | |
| 135 | 7 | F | UTI | S | S | R | R | S | R | R | R | UT | - | - | + | | - | | | - | | | + |
| 136 | 79 | M | UTI | R | R | R | S | S | R | R | R | O102 | + | + | + | | - | | | + | | | - |
| 137 | 51 | F | Cystitis | R | R | I | R | R | R | S | S | ROUGH | + | + | - | | + | | | + | | | - |
| 138 | 47 | F | Pyelonephritis | I | R | R | R | R | S | R | R | O95 | - | - | + | | + | | | - | | | - |
| 139 | 32 | F | UTI | S | I | S | S | S | R | R | S | O102 | + | - | - | | + | | | + | | | - |
| 140 | 34 | F | Pyelonephritis | I | R | I | R | R | S | R | R | UT | - | - | + | | - | | | + | | | - |
| 141 | 83 | M | Prostatitis | R | S | S | R | S | R | S | S | O1 | - | + | + | | + | | | + | | | - |
| 142 | 7 | M | UTI | S | I | S | R | R | R | R | R | O100 | + | - | - | | + | | | + | | | - |
| 143 | 56 | F | UTI | S | S | S | R | S | S | R | S | O1 | - | - | + | | - | | | - | | | - |
| 144 | 35 | F | Pyelonephritis | S | S | R | R | S | R | R | R | O102 | - | + | + | | + | | | + | | | + |
| 145 | 45 | F | Cystitis | R | S | R | R | R | S | R | R | NEC | + | - | + | | - | | | + | | | - |
| 146 | 1 | M | PUO | S | S | S | R | S | R | R | R | O102 | + | - | + | | - | | | + | | | - |
| 147 | 34 | F | UTI | I | R | S | R | R | R | R | R | O102 | - | - | - | | + | | | - | | | - |
| 148 | 65 | M | Prostatitis | S | S | R | R | R | R | R | R | O102 | - | - | + | | + | | | + | | | - |
| 149 | 29 | M | UTI | S | S | R | S | R | S | R | S | O102 | + | - | - | | + | | | + | | | - |
| 150 | 82 | F | Pyelonephritis | R | R | R | S | S | S | S | R | O102 | - | - | - | | + | | | - | | | + |

**Abbreviations**: UTI: urinary tract infection; PUO: pyrexia of unknown origin. (1) antibiotic sensitivity profile; CF: ciprofloxacin, NF: nitrofurantoin, Ca: ceftazidime, A: amoxycillin, NA: nalidixic acid, T: tetracycline, CO: co-trimoxazole, G: gentamicin (2)MSHA:mannose sensitive haemagglutination (3)MRHA: mannose resistant haemagglutination (4)HLY: hemolysis (5)SR: serum resistance (6)CSH: cell surface hydrophobicity (7)ESBL: extended spectrum beta-lactamases.
